# Teaching Craft Intelligence: Sovereignty Protocols for AI‐Mediated Student Leadership

**DOI:** 10.1002/yd.70070

**Published:** 2026-09-17

**Authors:** Christine Haskell

**Affiliations:** ^1^ Dative.works Dative.works Seattle, WA USA

## Abstract

Artificial intelligence (AI) now mediates how student leaders learn and decide, creating a speed imperative that often rewards algorithmic fluency over human judgment and intercultural accountability. This article argues student leadership programs can turn AI from a plagiarism risk into a laboratory for craft intelligence (transforming knowledge and lived experience into sound judgment in action) and deliberate stewardship, which safeguards how knowledge is created, tested, and shared. It maps four perils (e.g., inquiry collapse, context erasure, algorithmic authority, and cultural narrowing) that introduces a Trace of Agency toolkit of four sovereignty protocols to make AI‐assisted decisions transparent and leadership claims auditable.

## 1 AI as a Laboratory for Judgment

Artificial intelligence (AI) now mediates how student leaders learn, communicate, and decide, turning what once felt like a narrow “academic integrity” concern into a broader question of how judgment, authorship, and legitimacy are formed. Consider a student government president who asks an AI tool to draft a statement after a campus hate incident; within minutes, they have a calm, inclusive message that sounds right, but it misstates what happened, centers institutional risk, and omits the language local organizers have asked for. The risk in AI‐mediated leadership is not merely plagiarism, but what Hannah Arendt (1958) termed “rule by Nobody,” a bureaucratic state where automated processes replace human accountability. When student leaders defer to algorithmic outputs, they risk transitioning from Action (engaging in a shared public reality) to Labor (the mere survivalist production of content), causing the “intellectual muscles” required for critical judgment to atrophy.

Rather than resolving the conflict between automated efficiency and human judgment, Craft Intelligence functions as a practice of sustained tension. As long as this friction exists, the student maintains a *Trace of Agency*. If either the Arendtian “Nobody” or the Sennettian “Craftsman” wins outright, the human leader loses the capacity to navigate the ethical complexities of the digital age. This article argues student leadership programs can turn AI from a plagiarism risk into a laboratory for cultivating c*raft intelligence* and d*eliberate stewardship*. This shift is urgent because AI now touches the very capacities leadership education has promised to develop (e.g., communication, collaboration, and critical thinking) (Komives and Wagner 2012; Kuh 2008) and risks automating or bypassing them in favor of speed and polish when educators are not actively shaping its use (Susskind and Susskind 2015).

As technology firms rapidly define AI's capabilities without sustained input from educators (RAND Corporation 2023), accelerated adoption can quietly reconfigure how students learn to exercise judgment, authorship, and accountability in their leadership work, making companies the indirect stewards of cognitive development rather than educators. Treating AI as a laboratory for craft intelligence and deliberate stewardship is one way to keep those capacities central rather than incidental to leadership education.

### 1.1 Craft Intelligence and Deliberate Stewardship

This article responds to these conditions with two working constructs: *craft intelligence* and *deliberate stewardship*. Craft intelligence is the ability to turn knowledge and lived experience into sound judgment and creativity in action, including when AI assists with planning, drafting, or analysis (Sennett 2009; Adamson 2021). Deliberate stewardship is the responsibility to safeguard how knowledge is created, tested, and shared, so that leadership work remains accountable to human values, communities, and intercultural commitments rather than to what is easiest to automate or optimize (Lynch 2023). In AI‐saturated environments, craft intelligence functions as a pathway for students to develop and maintain agency with complex digital tools, rather than outsourcing judgment or authorship to them.

In student leadership contexts, these constructs shift the question from “Is this AI use allowed?” to “How was this decision made, whose knowledge did it draw on, and why should anyone trust it in this outcome?” The focus is on cultivating student agency with AI assistants while installing legitimacy guardrails that keep authority earned, situated, and reviewable instead of granted by fluency or platform visibility alone.

### 1.2 What This Article Offers

Recent *new directions for student leadership* (NDSL) work has also begun articulating AI‐enhanced leadership development frameworks grounded in equity and critical pedagogy (e.g., the Leadership Tree Model; Yang and Jimenez‐Luque 2025); this article complements that direction by offering sovereignty protocols that make AI‐assisted leadership claims auditable in practice. Aligned with NDSL's emphasis on applied, critical, and intercultural perspectives, this article provides a practice‐oriented contribution to the field of sociotechnical leadership. While craft intelligence is broadly applicable, these sovereignty protocols are specifically designed for secondary and post‐secondary leadership contexts where students engage in high‐stakes project‐based Action (e.g., student government, project management, community organizing, and policy drafting).

Rather than a mandate for every interaction, this framework offers a trace of agency for high‐stakes deliverables where human judgment is a professional and ethical requirement. It demonstrates how leadership programs can transcend the narrow focus on plagiarism to instead create structured environments for teaching deliberate stewardship. By embedding these four protocols into existing curricula, educators move students beyond passive consumption toward a Sovereign Making process where the human “Who” remains the governing force behind the algorithmic output.

### 1.3 Conceptual Framework

The archetypes presented in this article (The optimizer, extractor, framer, and preservationist) are not mere characterizations of student deficit; they are adaptive stances modeled after behavioral patterns ubiquitous in social media and algorithmic work environments (Gillespie 2018; Smith and Smith 2024). These stances emerge from the platform logic of modern AI, which incentivizes speed, visibility, and stochastic synthesis over contextualized judgment. By treating these archetypes as responses to structural pressures rather than fixed identities, educators can pivot from policing behavior to cultivating what Richard Sennett describes as the “basic human impulse to do a job well for its own sake (2009).”

To analyze these stances, I use two primary lenses:

- *Production* workflow (how work is made and whether students can show it)
- *Applied practice* (how work earns trust with specific audiences, standards, and consequences)

By utilizing these lenses, educators can plug the Proof of Thought toolkit into existing courses and programs so that AI‐assisted decisions leave a visible trail. This ensures that student work remains contestable, allowing the underlying reasoning to be questioned and evaluated as legitimate leadership work.

## 2 Perils in AI‐Mediated Leadership Roles and Work

In the opening vignette, where a polished AI output misstates events and erases organizer language, each peril below names a different way that judgment, authorship, and intercultural accountability can be displaced by platform‐speed defaults. The problem is not merely that speed produces mistakes; AI‐mediated fluency can skip the authentication leadership owes its publics (e.g., verifying what happened, locating whose language carries authority, and making standards for uptake explicit). When judgment is offloaded to algorithmic defaults, output becomes easier to generate than to warrant and *sounding right* can substitute for being answerable (Avery 2025). This section uses the production workflow and applied practice lenses to diagnose four perils and route them through the Proof of Thought toolkit.

### 2.1 Inquiry Collapse and the Optimizer

Under engagement‐optimized business models now face public scrutiny. Leadership education cannot treat AI outputs as neutral convenience, and must teach warranted judgment, or legitimacy collapses into what optimizes engagement (Grabenstein 2026). *Inquiry collapse* is a symptom of strategic credentialism, the rationalization of effort‐to‐reward ratios under conditions of extreme cognitive load (Smith and Smith 2024). In this stance, the student functions as the Optimizer, arriving at answers without the labor of understanding. They utilize generative AI as a high‐speed heuristic to achieve correct‐sounding outputs while minimizing treating AI outputs not as starting points for analysis but as pre‐vetted solutions.

Under the production‐workflow lens, this bypasses the slow work of framing questions and triangulating sources, allowing algorithmic fluency to replace visible reasoning (Mackenzie & Bhatt 2020; Gillespie 2018). In applied practice, the *Optimizer* equates “looking right” with earning trust, importing platform‐driven cues such as length, tone, and formatting as proxies for genuine legitimacy. By offloading the labor of original schema formation, the Optimizer adopts the algorithmic incentive structures of digital platforms, where speed and formatting supersede epistemic depth as the primary metrics of authority.

In an educational context, inquiry collapse represents a structural bypass of foundational cognition. By generating high‐fidelity outputs instantaneously (i.e., a polished business vision or mission statements for a leadership course), AI facilitates a “Bloom's Shortcut” that omits prerequisite learning phases of remembering, understanding, and applying nascent knowledge and expertise (Bloom et al. 1956; Anderson and Krathwohl 2001). Such cognitive offloading atrophies the intellectual, reasoning, and moral creativity muscles required to build the deep mental schemas underpinning expert judgment (Arendt 1973; Avery 2025). The result is a *hollowed‐out fluency*: a polished performance the student cannot epistemically challenge or adequately defend, reinforcing a false leadership posture prioritizing terminal output over the traceable reasoning required for genuine stewardship. In leadership terms, inquiry collapse produces authority without ownership: a student can issue a polished public message yet cannot name its warrants, explain the trade‐offs behind it, or revise it responsibly when stakeholders contest it (Christin 2020; Andrejevic 2019).

### 2.2 Context Erasure and the Extractor

*Context erasure* occurs when the Extractor utilizes AI to divorce knowledge from its lineages, relationships, and histories (Beer, 2022). This adaptation solves the local problem of synthesizing collaborative inquiry or community input into solo output under pressure. From a production workflow standpoint, this approach hides the social, intellectual, and material labor (often in the Global South) required to train the models that produce these artifacts; AI becomes a “black box” that ingests prompts and produces prose, while original contributors recede into generic acknowledgments or disappear altogether (Christin 2020; Andrejevic 2019). Through the lens of applied practice, extraction allows leaders to claim authority without visible provenance, asking audiences to trust outputs that cannot be traced back to the people, communities, or texts that could contest or refine them. In leadership terms, context erasure is how a community becomes a data source rather than a co‐author: the leader inherits language without consent, provenance, or a repair pathway when those most affected contest the framing (Gillespie 2018; Noble 2018).

### 2.3 Algorithmic Authority and the Framer

*Algorithmic authority* emerges when rankings, virality, and visibility substitute for disciplinary or community standards (Gillespie 2018). In this mode, the student becomes the *Framer*, prioritizing platform‐friendly resonance over rigor. Seen through the production‐workflow lens, the Framer narrows inputs to top‐ranked sources or AI‐generated options, rarely documenting which nondigitized knowledge or communities were excluded from the dataset. In applied practice, this stance encourages leaders to equate visibility with credibility, treating “likes” and search prominence as evidence that a proposal works even when it sidelines less visible or prevalent stakeholders, or conflicts with ethical standards (Noble 2018; Mohamed et al. 2020). The Framer's reliance on platform‐friendly resonance over rigor mirrors Arendt's (1971) observation on the *quicksand of official fiction*. When visibility and engagement metrics replace disciplinary standards, the distinction between fact and opinion dissolves. This increases the public realm (and the student leaders who inhabit it), vulnerable to manipulation and the erosion of a shared reality, especially when fluent model outputs are mistaken for knowledge rather than for pattern‐completion over web‐scale corpora (Bender et al., 2021).

While framing is necessary for communication, the Framer archetype allows platform incentives or personal agendas to outpace the need for lineage and accountability. They excel at the aesthetic redressing that makes complex ideas accessible, illustrating how resonance and reach can outpace rigor and lineage when no counter‑practice requires them to name standards and audiences that do not map neatly onto engagement metrics.​ In leadership terms, algorithmic authority is legitimacy laundering: platform signals (rank, reach, engagement) stand in for standards, so leaders can't show why a claim deserves uptake when the audience asks, “By what right, and by whose criteria” (Smith 2012; Santos 2014; Mohamed et al. 2020; Lynch 2023)?

### 2.4 Cultural Narrowing and the Preservationist

*Cultural narrowing* occurs when information ecologies flatten toward Anglophone, digitized, and text‐centric norms. The student responds as the *Preservationist*, resisting AI adoption to protect craft, ethics, and local knowledge (Lynch 2023). However, when this stance becomes total withdrawal, it inadvertently reinforces the very narrowing it resists (Avery 2025). In production workflow, avoiding AI entirely can leave the technology to lower‐standard uses, ensuring that Indigenous, oral, and non‐Western epistemologies remain absent from the systems shaping the future, while in applied practice this retreat leaves critical perspectives underrepresented where decisions are actually being shaped (Smith 2012; Santos 2014; Mohamed et al. 2020). The goal is not withdrawal, but structured engagement that pushes plural sources into AI‐mediated workflows so that Preservationist impulses protect craft and ethics without ceding AI spaces altogether.

These archetypes are not fixed identities, but adaptive stances taken in response to institutional and platform pressures. Each offers a partial solution (i.e., efficiency, resonance, synthesis, or protection) but on their own, they cannot restore trustworthy knowledge. The toolkit that follows operationalizes the countermoves required to achieve *craft intelligence*: an integrated state where domain expertise remains non‐negotiable, and outputs are evaluated against transparency, plurality, and consequence literacy. In leadership terms, cultural narrowing isn't mere bias; it is a structural foreclosure of what can count as evidence, shrinking the epistemic field so that leadership *reasonableness* becomes indistinguishable from the platform's default canon.

### 2.5 Toward Craft Intelligence

The target archetype is the *Craftsperson*, though not precisely the one Sennett envisioned (Sennett 2009; Adamson 2021). This leader refuses to collapse the tension between the machine's speed and material's resistance. They do not seek to *resolve* the Arendtian peril of automation through simple labor; rather, they utilize the ethos of craftsmanship to *reclaim* the human “Who” that Arendt (1958) feared would be lost to the “Rule by Nobody.”

The preference for craft intelligence *(CI)* is a requirement for *epistemic sovereignty*. Sennett (2009) explicitly challenged Arendt's “dim view” of manual work, arguing the “desire to do a job well for its own sake” is not a mindless activity but the foundation of human well‐being and judgment. While Arendt (1958) worried about a focus on making (Work) would alienate the individual from leadership (Action), Sennett contended that the “head and the hand” are inseparable. In an AI‐mediated environment, this connection is the final line of defense: if the “hand” (the process of making) is outsourced to an algorithm, the “head” (the capacity for judgment) inevitably atrophies.

In this framework, *tension is the prerequisite for freedom*. This is a direct resistance against the “low‐standard / low‐tension” success models of current geopolitics, where efficiency is often mistaken for authority. Freedom is not found in the *Action* of a leader who merely delegates to an algorithm (Arendt's “Nobody”), nor is it found in *Labor* performed for mere reward. Instead, it is found in the *Craftsperson's struggle with the material*.

By introducing deliberate, constructive friction, the leader exercises *Pluralistic Freedom*: the liberty to exist outside the text‐centric “Optimizer” bubble and remain tethered to lived reality. Without these agency trails, these “marks of the maker,” leadership risks dissolving into a “quicksand” of automated fictions (Arendt 1973). Reaching this state requires repeatable structures (e.g., Sovereignty Protocols) that ensure the “intellectual and creative muscles” of judgment are defined by what human imagination can achieve rather than what a platform can generate.

## 3 From Perils to Practice: Sovereignty Protocols

This section introduces the Sovereignty Protocols, a Trace of Agency toolkit designed to help leadership educators reclaim human agency within AI‐mediated environments. Developed in conversation with Fields (2025) and Hurtado et al. 2025), they require a reviewable decision trail so AI‐assisted work earns legitimacy against explicit standards and audiences.

The four protocols that follow operationalize the Arendtian and Sennettian theories discussed in previous sections. Where Arendt warned against the “rule by nobody” and Sennett championed the craftsperson's commitment to process, these protocols translate those abstract philosophical concerns into daily student behaviors (Arendt 1958; Sennett 2009). Think of them as scaffolding that transforms Craft Intelligence from a theoretical ideal into a routine habit: each protocol functions as a *friction checkpoint* that prevents the automated default from erasing the human “Who” behind the decision. Together, they form a tangible, archivable system that makes AI‐assisted leadership work auditable, legitimate, and educationally defensible. These protocols treat opacity as a governance problem: when algorithmic systems shape public‐facing decisions, leaders must be able to show methods and warrants, not merely present outputs (Pasquale 2015).

In practical terms, the protocols below give instructors a way to require evidence of judgment and lineage care in exactly the kinds of high‐stakes communications introduced at the outset.

### 3.1 Implementation Structure and Culture: Cultivating Sovereignty

Implementing these Sovereignty Protocols requires balancing structural documentation (“hard practices”) with a culture of productive friction (i.e., “soft practices”). Without this cultural container, protocols risk devolving into mere compliance paperwork. To cultivate Craft Intelligence and move beyond the *Rule by Nobody*, educators must first scaffold an environment that prizes the human *who* behind the decision:

- **Modeling the Struggle**: Instructors should transparently share their own Decision Maps, revealing where they personally utilized AI, where the model failed, and where human judgment explicitly overrode the algorithm (Fields 2025; Arendt 1973; Sennett 2009; Yang and Jimenez‐Luque 2025).
- **Tangible Friction**: To prevent inquiry collapse, educators can require “tangible making” phases (e.g., paper storyboards or analog mapping) before students engage with an AI prompt. This forces original schema formation and asserts cognitive sovereignty before the “Optimizer” mindset, and its accompanying speed imperative, takes over (Sennett 2009; Bloom et al. 1956; Anderson and Krathwohl 2001; Smith and Smith 2024b; Hurtado et al. 2025; Keune et al. 2024).
- **Lineage Language**: Reframing citation as *deliberate stewardship* asks students to name the “ancestors” of an idea (e.g., texts, community elders, or specific datasets). This shifts the student's identity from a passive Extractor toward a Relational Accountability Leader who recognizes that all knowledge has provenance (Smith 2012; Santos 2014; COPE Council 2019; Mohamed et al. 2020).

For educators adopting this toolkit, we recommend a sequenced introduction rather than implementing all four protocols simultaneously. Begin with Decision Maps (DM) in early coursework to establish the habit of making reasoning visible and vetoing AI defaults. Once students demonstrate comfort with documenting their cognitive process, layer in the Lineage Ledger (LL) to build contributor accountability. In upper‐level or capstone projects, introduce the Legitimacy Check (LC) and Source Plurality Protocol (SPP) where students engage with external stakeholders and manage higher‐stakes decisions. This scaffolded approach allows students to develop sovereignty habits incrementally, preventing protocol fatigue while building toward full craft intelligence by graduation.

### 3.2 Decision Maps: Making Reasoning Visible

*Decision Maps* (DM) are one‐page sovereignty exercises that make trade‐offs visible in AI‐mediated decisions (Fields 2025). They dethrone algorithmic *fluency* by surfacing the human “Who” behind the choice. This is where the student faces the Arendtian *Void* (i.e., the silent, automated *default*) and applies Sennettian *Friction* through the Veto.

- **Purpose**: Assert cognitive sovereignty by documenting the reasoning behind a decision. This is where the student faces the Arendtian *Void* (the AI *default*) and applies Sennettian *Friction* through the Veto (Arendt 1973; Sennett 2009).
- **When to use**: Any AI‐assisted memo, proposal, policy draft, or campaign plan where students are choosing among options.
- **Inputs**: Minimum 3x discipline‐specific repositories and 2x local/community sources, alongside AI outputs.
- **Output**: A map documenting the Veto, the point where the leader rejected an AI suggestion. The tension between automation and agency is resolved only for this specific decision, then resets for the next inquiry.
- **Evidence generated**: Process Quality, Transparency, and Epistemic Agency.
- **Freedom Established**: The Freedom of Intellectual Sovereignty. This is the freedom to develop deep mental schemas and internal *maps* that cannot be manipulated or erased by external platform logic.

### 3.3 Legitimacy Check

A *Legitimacy Check* (LC) tests whether a claim deserves uptake by specific audiences. It anchors credibility in standards rather than platform visibility or AI‐generated rankings (Gillespie 2018; Noble 2018; Mohamed et al. 2020).

- **Purpose**: Anchor credibility in professional and community standards rather than platform visibility or AI fluency. This requires the student to move beyond the Arendtian “Nobody” of the algorithm by identifying the specific stakeholders and standards to which they are personally accountable (Arendt 1970; Gillespie 2018).
- **When to use**: Any AI‐assisted recommendation, policy, or public communication where student leaders ask others to act on their claims.
- **Inputs**: Audience criteria, evidence thresholds, and a stakeholder impact scan.
- **Output**: A justification of why a recommendation holds weight even when platform signals are set aside. The LC represents the Sennettian craftsman's commitment to “doing a job well for its own sake” by prioritizing audience‐specific truth over algorithmic consensus (Sennett 2009).
- **Evidence‐generated**: Legitimacy and Consequence Literacy. It proves the leader's “Who” is bound to the consequences of the claim within a real‐world context.
- **Freedom Established**: The Freedom of Accountability. This is the freedom to exist as a responsible “Who” at the center of a decision, reclaiming the right to be held answerable to a human community rather than a statistical probability.

### 3.4 The Lineage Ledger (LL)

The *Lineage Ledger* (LL) ensures timeline and labor are explicit, preventing the “Rule by Nobody” by naming exactly who, human or machine, authorized the text (COPE Council 2019).

- **Purpose**: Make intellectual lineage and labor visible by formalizing textual, nontextual, and communal contributions. It serves as a Sennettian “mark of the maker,” preventing the Arendtian “Nobody” from absorbing the student's unique labor.
- **When to use**: Any collaborative report, public statement, or creative artifact where AI generation, community input, or multiple authors are synthesized into a single voice.
- **Inputs**: Contributor roles (human/AI/community), contribution modes (e.g., oral, observational, analytic), and relevant consent or permissions.
- **Output**: A contributorship table and methods note providing a Warrant of Agency. As the ultimate proof of the “Who,” the Ledger forces the student to stand behind the work, preventing the Arendtian “Nobody” from taking credit for the leadership claim.
- **Evidence generated**: Transparency, Plurality, and Contributorship Accountability.
- **Freedom Established**: The Freedom of Authorship. This is the freedom to claim one's own intellectual history and labor, ensuring the leader is not just a consumer of outputs but a recognized steward of knowledge.

### 3.5 Source Plurality Protocol (SPP)

The *Source Plurality Protocol* (SPP) is a systematic audit of absence. For Western audiences, this transforms a theoretical ideal into a routine practice of epistemic humility. By making the cultural narrowing of the model visible, the leader ensures that plural epistemologies, rather than just data‐rich corpora, authorize the final decision (Smith 2012; Santos 2014; Mohamed et al. 2020). This also treats training‐data opacity and hegemonic overrepresentation as a reason to require plural, situated sources alongside AI summaries (Bender et al., 2021).

- **Purpose**: Make it routine to test AI against nondominant, local, and nontextual knowledge. This balances the Arendtian risk of “Cultural Narrowing” against the Sennettian craft of “Sourcing” (Arendt 1970; Sennett 2009).
- **When to use**: Any research phase or environmental scan where AI‐generated summaries are used to define the history or scope of a challenge.
- **Inputs**: Paired sets of sources (AI/top‐ranked vs. local/oral/nonAnglophone), plus brief notes on agreements and conflicts.
- **Output**: A short log documenting where sources align or diverge. This ensures the leader's “Who” is informed by more than just the dominant algorithmic consensus (Santos 2014).
- **Evidence generated**: Epistemic Humility and Intercultural Plurality.
- **Freedom Established**: The Freedom of Plurality. This is the freedom to think and decide outside the consensus of the data model, maintaining a vital tether to lived, local, and diverse realities.

### 3.6 Examples of Non‐Dominant Sources for Educators

To operationalize plurality in practice, instructors should guide students toward these specific source types:

- **Oral Histories**: Recorded interviews with community elders, organizational founders, or local activists (e.g., StoryCorps archives, university oral history collections).
- **Community Archives**: Non‐digitized meeting minutes from neighborhood councils, tribal governments, or grassroots organizations.
- **Local Knowledge Repositories**: City planning documents, regional environmental impact studies, or municipal public comment records.
- **Non‐Anglophone Sources**: Scholarship published in languages other than English, particularly from the Global South (e.g., Latin American, African, or Asian journals).
- **Practice‐Based Knowledge**: Craft manuals, apprenticeship documentation, or embodied knowledge from practitioners (e.g., union organizers, artisans, social workers) Indigenous Epistemologies: Traditional ecological knowledge.
- **Indigenous** land management practices, or community‐specific protocols (always with appropriate permissions and acknowledgment).

By pairing these sources with AI‐generated or top‐ranked results, students surface what the algorithm excludes and practice the epistemic humility required for decolonized leadership.

Consider a student leader drafting a community partnership memo. An *Optimizer* prompts AI and submits a generic statement: fluency without relationship. A *Steward* uses the SPP to check the AI's history against a local tribal archive. They record a “Veto” where the AI used colonial phrasing. While the final memos may look similar, the Steward's “Trace of Agency” packet proves they exercised cognitive sovereignty, maintaining the productive tension that is the hallmark of human freedom. Toolkits can be layered for high‐stakes decisions (see Table 1).

**TABLE 1:** The Sovereignty protocol framework.

Analytical lens	Protocols	Primary function	Strategic objective
Production workflow	DM & LL	Tracing inputs, methods, and labor	Sovereign *making*
Applied practice	LC & SPP	Testing claims and epistemic plurality	Legitimate *doing*

## 4 Program and Assessment Implications

Embedding this toolkit in student leadership work gives programs a way to make deliberate stewardship visible. By utilizing the sovereignty packets described in Section 3.1, instructors shift assessment from performative fluency to the quality of reasoning, lineage care, plural sourcing, and consequence literacy.

### 4.1 Assessment: From Detection to Development

To move beyond policing AI use, educators require assessment tools that distinguish performative fluency from actual judgment consistent with leadership development approaches that emphasize reflective, equity‐centered practice (Yang and Jimenez‐Luque 2025). Table 2 below outlines a rubric for Craft Intelligence that aligns with the adaptive stances explored earlier. By allocating points for transparency and process quality, this framework rewards the “friction” generated by critical thinking while identifying where students may be drifting into the passive role of Optimizer (Sennett 2009; Anderson and Krathwohl 2001).

**TABLE 2:** Rubric for assessing craft intelligence and deliberate stewardship.

Criteria	Inquiry collapse / strategic optimization (Minimal viable performance)	Proficient practice (Competent)	Craft intelligence / The steward (Exemplary)
**Transparency** (Fields 2025; Arendt 1973; Sennett 2009)	The decision trail is opaque; relies on “it felt right” or AI fluency. Methods are obscured.	The decision trail is visible but may lack detail on specific inputs or prompt iterations.	**Complete and followable**. The Decision Map fully traces inputs, disciplinary methods, and the specific human judgment applied to the final call.
**Process Quality** (Sennett 2009; Bloom 1956; Anderson and Krathwohl 2001; Smith et al. 2024))	Accepts the first feasible solution (usually the AI default). No distinct alternatives or trade‐offs shown.	Considers alternatives but selects based primarily on efficiency or surface‐level compliance.	**Reflects field constraints**. Explicitly documents discarded options, trade‐offs, and why specific alternatives were rejected based on disciplinary values.
**Plurality** (Smith 2012; Santos 2014; Mohamed et al. 2020)	Relies on “top‐ranked” or AI‐generated corpora. Cultural narrowing is evident.	Consults multiple sources but they remain within the dominant/digitized canon.	**Triangulation**. Systematically pairs AI/dominant sources with non‐dominant, oral, or community‐based sources (via the Source Plurality Protocol).
**Legitimacy** (Gillespie 2018; Noble 2018)	Claims authority based on tone, polish, or format. Assumes visibility equals validity.	Claims are supported by general evidence but may miss context‐specific audience needs.	**Audience Fit**. Claims are tested against specific evidence thresholds and audience standards (via Legitimacy Check). “Fit” is explicit and warranted.
**Consequence Literacy** (Mohamed et al. 2020; Noble 2018)	Focuses on the immediate solution. Risks are ignored or treated as *technical glitches*.	Identifies obvious risks but relies on standard disclaimers rather than structural changes.	**Anticipatory**. Risks and harms (e.g., exclusion, bias) are anticipated, and specific revisions/safeguards are documented in the final artifact.

*Note*. Criteria are author‐developed; protocol terms are defined in Sections 3.2–3.5; conceptual grounding draws on Arendt/Sennett (craft, judgment), decolonial and epistemic justice scholarship (plurality), and platform/algorithmic authority scholarship (legitimacy).

This rubric operationalizes the *proof of thought* packet by shifting assessment from the polish of the output (which AI can mimic) to the visible reasoning of the process. The *Craft Intelligence* column maps to widely recognized leadership capacities (e.g., critical thinking (process quality), ethical reasoning (consequence literacy), intercultural competence (plurality), and communication (transparency), so instructors can justify process‐based grading within existing program outcomes.

At the program level, these compact sovereignty packets function as archivable evidence that leadership education is cultivating judgment, collaboration, and intercultural accountability under AI‐saturated conditions. By requiring these artifacts alongside key assignments, programs generate direct evidence that students are practicing the communication, critical thinking, and intercultural judgment that leadership curricula and high‐impact practices promise to develop (Association of American Colleges and Universities [AAC&U], 2013; Kuh 2008; Komives and Wagner 2012). This moves program reviews beyond output‐only assessments, offering reviewers a robust picture of how decisions were made and whose knowledge shaped them.

### 4.2 The Institutional Environment: The Sovereignty Clause

The sovereignty clause treats expertise as something institutions must defend through rules of accountability, not merely as an individual virtue (Pasquale 2020). For Craft Intelligence to move from a classroom‐scale exercise to a sustained leadership practice, it requires an institutional infrastructure that rewards constructive friction over algorithmic fluency. This necessitates a “Sovereignty Clause” in student handbooks: policy language that explicitly authorizes AI use *provided* the student submits a Trace of Agency packet (DM, LL, LC, or SPP) as evidence of cognitive sovereignty. Such a clause moves the institution from a posture of detection (plagiarism policing) to development (stewardship), ensuring that the “intellectual and creative muscles” of judgment are protected by governance rather than just personal preference.

Sample Sovereignty Clause Language for Student Handbooks:

AI tools may be used for leadership assignments when accompanied by a Trace of Agency packet (Decision Map, Lineage Ledger, Legitimacy Check, and/or Source Plurality Protocol) documenting how human judgment governed the work. For high‐stakes deliverables (e.g., public statements, policy proposals, strategic plans), at least one protocol is required. Submissions without a packet may be returned for revision.

This institutional framing transforms AI policy from prohibition to pedagogy, signaling to students, faculty, and accreditors that the institution values process integrity alongside product quality.

## 5 Holding the Line on Knowledge Stewardship

Ultimately, teaching Craft Intelligence is an effort to sustain intellectual, moral, and creative muscle memory in an age of automation. This is not mere enrichment; *it is the cognitive and societal infrastructure* required to resist the “quicksand” of automated fictions Arendt (1973) warned would leave society vulnerable to manipulation. The task is not only to “use AI responsibly,” but to govern the terms under which knowledge is made, shared, and trusted.

The Craftsperson archetype is not a leader who has “solved” the AI problem, but one who has learned to inhabit the permanent tension between the speed of the machine and the resistance of the material. Through these protocols, student leaders restore the link between the “head and the hand” that Sennett (2009) identifies as the core of human well‐being. These practices function as counterweights to systemic forces by operationalizing four concrete mechanisms.

- Decision Maps (DM) and Lineage Ledger (LL) to make the *making* reviewable, and
- Legitimacy Checks (LC) with a Source Plurality Protocol (SPP) to make the *doing* worthy of trust.

Used together, these tools transform learning into a visible, relational governance of ideas. Students show their reasoning, restore lineage, and widen their evidence base, while programs gain archivable artifacts that demonstrate learning for accreditation and review.

When these practices are sustained in climates of uncertainty, learners grow into leaders capable of integrating methods with judgment and efficiency with rigor. In this framework, the tension itself is the source of (epistemic and moral) freedom; it ensures that judgment remains defined by what human imagination can achieve rather than what a platform can generate.
